# Supplementary material for: Flywheel eccentric overload exercises versus barbell half squats for basketball players: Which is better for induction of post-activation performance enhancement?
Source: PLoS One. 2022 Nov 21;17(11):e0277432. doi: 10.1371/journal.pone.0277432 (PMC9678314; doi:10.1371/journal.pone.0277432)
Supplement: Attachment — Submitted filename: Response to Reviewers.docx [file pone.0277432.s002.docx]

**Dear editor and reviewers**

Regarding the manuscript ID (NO.PONE-D-22-02783) entitled " Eccentric overload exercises versus loaded half squats for basketball players: which is better for induction of postactivation potentiation?". Thank you for your letter and the reviewers' comments on our manuscript entitled " Eccentric overload exercises versus loaded half squats for basketball players: which is better for induction of postactivation potentiation?" (NO.PONE-D-22-02783) were evaluated. These comments were valuable and helpful to us in revising and improving the paper, as well as important guidance for our research. We have carefully studied these comments and made revisions, which we hope will be approved by all of you. The revised parts are marked in red in the thesis.and The main corrections in the paper and the responses to the reviewers are listed below.

**Responds to Reviewer #1:**

**1. Response to comment:** *L 52:Undefined previously, please define.*

**Responds:** We greatly appreciate the reviewers' comments. We have changed the definition of PPO to Peak power output (PPO). In L52

**2. Response to comment:** *L 54-55:"Could increase PPO to a certain extent" is an inapropriate statement to present results. Please rephrase or present the magnitude of this increase.*

**Responds:** Thank you for your valuable comments. For the reviewers' valuable comments, we have revised the content to EOL training in the second stage increased PPO to a certain extent, especially at 3 min (p=0.046, ES=0.716). In L56-58

3**. Response to comment:** *L 58:Performances were only assessed at different precise timing after exercise, and not during different period after exercise termination. Therefore, I suggest using time-points rather than time windows.*

**Responds:** Thank you for your valuable comments. For the valuable comments of the reviewers. We have changed the time windows to time-points. In L63-64

**4. Response to comment:** *L 67-70:Please conform to the findings of the cited reference (and others e.g.; Blazevich and Babault, 2019; DOI: 10.3389/FPHYS.2019.01359), by mentioning the difference between electrically-evoked and voluntary improvements in performance.*

**Responds:** We are very grateful for the reviewers' comments. For the reviewers' valuable comments, we add the study by Blazevich and Babault. In this study, the term PAPE was used in relation to the enhancement of muscle contraction after conditioning activity. In L80-81

**5. Response to comment:** *L 72-74:Only referring to phosphorylation of MHC or change in fascicle pennation angle is really scarce in light of others potentials factors driving improvement in voluntary performance (e.g. muscle temperature, muscle blood flow), and particularly when focusing on eccentric mode of muscle action (e.g. neural factors). Please amend to provide a reliable overview of these mechanisms.*

**Responds:** We are very grateful for the reviewers' comments. After carefully reading the reviewers' comments, we have added the concepts and mechanisms related to PAP and PAPE. In L83-90.

**6. Response to comment***: L 78:Typing error : provided*

**Responds:** Thank you for your valuable comments. After carefully reading the reviewers' comments, we have changed the typing to provided.

**7. Response to comment:** *L 80-81*:*Because squat exercise modality can induce a PAPE only in the lower limbs (in comparison to the upper limbs), I suggest the authors to precise this element in order to avoid misunderstanding by novice readers.*

**Responds:** We greatly appreciate the reviewers' comments. For the reviewer's valuable comment, we have changed it to Squat exercise can induce a PAPE in the lower limbs. In L94.

**8. Response to comment:** *L 82-83:Please precise which parameters were improved in these studies (e.g. jump height, rate of force development, reaction time, maximal power, maximal speed, ...). Indeed, the conflicting findings about the factors improved by PAPE which depend upon the conditioning sequence should be avoid here.*

**Responds:** Thank you for your valuable comments. After carefully reading the reviewers' comments, we have listed the relevant metrics: strength, power, sprint, jumps and maximal voluntary contraction (MVC). In L100.

**9. Response to comment:** *L 85:"Has been pro" should be rephrase for a better understanding*

**Responds:** Thank you for your valuable input. After carefully reading the article, we have changed to Heavy and moderate weighted squats (90% and 60% of 1RM, respectively) have been reported to improve sprint and CMJ performance in male subjects. In L97

**10. Response to comment:**. *L 96-100:Please also report the above-mentioned study (i.e. Norrbrand 2010) that reported similar improvement in MVC than the concentric training.*

**Responds:** Thank you for your valuable input. After carefully reading the article, we have made revision and addition in the corresponding places.

**11. Response to comment:** *L 101-103:Although scarce, please cite some references focusing on jumping performance (e.g. French et al., 2003, DOI: 10.1519/ 1533-4287(2003)017<0678:CIDEPF>2.0.CO;2)*

**Responds:** Thank you for your valuable comments. After carefully reading the reviewers' comments, we have described their impact on jump performance and added relevant references. In L114-116.

**12.Response to comment:** *L 116:It is unclear what are the short-term effects mentioned here. The authors stated a two-stages randomized cross-over study, lasting for 13 weeks.*

**Responds:** We are very grateful for the reviewers' comments. We have rewritten the sentences to compare the acute effects of the EOL protocol (three intensities) and the HS protocol (three intensities) on CMJ jump height or 30 m sprint performance after each training session(each intensity). In L129-131

**13.Response to comment:** *L 116:The greater sprint performance could only be speculated here, and could therefore be mentioned as an expected finding at the end of the introduction section.*

**Responds:** We are very grateful to the reviewers for giving such a high rating to our article. We have rewritten the Experimental approach to the problem in order to be more clear about the design of the experiment and in response to the question you raised. In L124-140

**14.Response to comment:** *L 129:Rephrase as : and they were then informed about the EOL protocol.*

**Responds:** After reading the article carefully, we have rewritten the sentence: They were then informed and familiarized with the EOL and HS protocols, CMJ jumps and 30m sprint tests. In L173-175.

**15.Response to comment:** *L 130-131:Please provide additional explanations about the expected effects of the familiarization procedure for experienced subjects.*

**Responds:** Thank you for your valuable suggestions. After carefully reading the reviewers' comments, we have added the following clarification: Participants in this trial typically performed resistance training in the strength and conditioning laboratory once a week as a training task, and therefore had a priori knowledge of the training protocol and testing methods. An additional familiarization exercise was provided prior to the experiment. In L158-160.

**16.Response to comment:** *L 133-139:The procedure implemented to assess CMJ and sprint performance requires further supporting elements. Could the authors indicate for instance why they did not alternate the testing between CMJ and sprint?*

**Responds:** We are grateful to the reviewers for their comments. We are currently hypothesizing in accordance with your opinion that participants did not alternate CMJ and sprint tests during the first 3 sessions based on the hypothesis that cross-testing after one training session (particularly in the EOL protocol) may induce some muscle damage that could lead to biased maximal performance at CMJ or sprint. We also tried to further improve the reliability of our conclusions by conducting the same repeated trials based on the same group of investigators at different time periods. In L144-147.

**17.Response to comment:**. *L 139:Please add the time-delay allowed between the two sequences of this protocol.*

**Responds:** We are very grateful for the reviewers' comments. We would like to express the meaning: Training break of 1 week (14 days) between the two programs. In L141-144.

**18.Response to comment:***Also, justify that the 1-week washout period is sufficient to alleviate the effects of the first protocol on the testing and effectiveness of the second sequence. (which seems unlikely).*

**Responds:** We greatly appreciate the reviewers' comments. We are currently revising in accordance with your comments to: The authors interrupted the training sessions for 1 week as a washout period (14 days between training sessions) to mitigate the effects of the first protocol on the second protocol tested further research was required. In L141-144.

**19.Response to comment:***L 149:I recommend the authors to avoid the excessive use of decimals and report the appropriate precision provided by the measurements they performed.*

**Responds:** We greatly appreciate the reviewers' comments. We have added the table about the basic information of the participates. (age, height, weight, etc.) to the attachment. L 260-265

**20.Response to comment:**L 161:*Should be : "subjects were asked to"*

**Responds:** Thank you for your valuable comments. We have revised the presentation. All subjects were informed about the potential risks and benefits of the current procedures and were asked to signed an informed consent form. In L162.

**21.Response to comment:**L 170:*Please detail what are those "basic information"*

Responds: We are very grateful for the reviewers' comments. We have added the table about the basic information of the participates. (age, height, weight, etc.) to the attachment. L 260-265

**22.Response to comment:**. L 178-180:*Do the authors control for the reproducibility between the two trials to ensure the validity of the calculated mean? If so, please provide the accepted range between trials to ensure two maximal performance.*

**Responds:** We greatly appreciate the reviewers' comments. We have added the motion control method between the two trials: All test and training interventions are set up with the appropriate movement control mode. Technical feedback from the field is provided by experienced investigators. Tests and training sessions are re-executed once the subject's movements are incorrectly executed or not at maximum effort. In L178-182.

**23.Response to comment:**L 181: *What was the recovery modality allowed to the subjects between the different time points?*

**Responds:** We are grateful for the reviewers' comments. We added the recovery method for different time points and the participants used passive recovery between time points. In L191-192.

**24.Response to comment:** *L 191-193:Please provide the height of the photoelectric cells.*

**Responds:** We greatly appreciate the reviewers' comments. We have added the height of the equipment: These photocells were mounted 1 m above floor level. In L207.

**25.Response to comment:***L 205:In accordance with one of my previous comment regarding the lack of details about the "basic information" recorded by means of body scale, the authors should provide additional information regarding the method used to calculate body surface area.*

**Responds:** Thank you for your valuable comments. We appreciate the reviewers' comments. We have added the basic information to the attachment. We are very sorry that our Inbody370 device was not able to provide body scale calculations.

**26.Response to comment:** *L 207-209:Please precise whether the pace was controlled during the downward phase of the half squat. If so, this information should be added to provide an estimate of the braking resistance applied by the subjects.*

**Responds:** Thank you for your valuable comments. We have added the motion control method. Subjects started in a half-squat position and were asked to execute the concentric phase as quickly as possible and to control the braking phase until the knees were flexed to approximately 90 degrees. In L219-222.

**27.Response to comment:** *L 214-215:It seems that the amount of repetitions per set is lacking here. Please amend.*

**Responds:** Thank you for your valuable comments. We have modified the number of reps. For performing 5 sets × 3 reps with a 2-minute rest period between sets. In L229.

**28.Response to comment:** *L 225-227:Provide further explanations about the meaning one his one-way ANOVA. Indeed, your protocol* *implemented two exercises and three modality. How could the authors therefore performed only a one-way ANOVA?*

**Responds:** We apologize for any confusion caused in the article. In our design, different intensity(3 intensities) was implemented each week for three weeks, and repeated measures of outcome indicators were performed after each trial. Given the specificity of the trial, we viewed the three intensities as independent of each other and therefore performed a one-way repeated measures ANOVA in each intensity. We also did a two-way ANOVA. We have uploaded the statistics in the attachment and hope it will help you to solve the confusion of reading. L 241-256

**29.Response to comment:** *L 233-234:Statistical information are lacking in figures (significant different and not only ANOVA F or p values) forcing the readers to back-and-forth between figures and text. It could be therefore suitable to add the calculated 95%CI on the graphs and symbols to indicate statistical differences.*

**Responds:** Thank you for your valuable comments that would enhance the quality of our manuscript. We have tried as much as we can, however, due to the dense graphics, the graphics appear more confusing after adding 95% CI, which seriously affects the clarity of the graphics. For this reason, we weighed again and again and apologized for not adding 95% CI to the graphics. We have uploaded the statistics in the attachment and hope it will help you to solve the confusion of reading.

**30.Response to comment:** *L 240-243:The comparisons between time points and intensity for pre-HS and pre-EOL is unclear here. It is state in the protocol that each session differed from the other by the testing protocol (HS or EOL) or the intensity (low, med and high). Please amend therefore to understand which variables were compared here.*

**Responds:** Thank you for your valuable comments that so enhance the quality of our manuscript, and We have revised the study design of the manuscript, which we hope will contribute to the understanding of the results. In the first phase (weeks 1-6) participants were randomized into two groups (pre-EOL vs. pre-HS) and in the second phase (weeks 8-13) participants crossed over between the two groups (pre-EOL to post-HS, pre-HS to post-EOL). We also have revised flowchart of the experiment. and re-uploaded it as an attachment. In L126-129

**31.Response to comment:** *L 249: Could you please indicate the degree of freedom for the F value of the ANOVA. In addition, I suggest to avoid the appelation of week1, 2 or 3, should rather precise the intensity that is one of the main independent variable.*

Responds: Thank you for your valuable comments that so enhance the quality of our manuscript, and We have made revisions and added information to the manuscript in the appropriate places based on the comments.

**32.Response to comment:** *L 252-253:This sentence requires rephrasing to avoid misinterpretation. Under the current version, this sentence would means that the performance at PRE is significantly related to measurements made at POST. However, it seems that this analysis was not performed, and is not of primary importance for this study.*

**Responds:** We apologize for any confusion caused in the article. We used a two-stage crossover design. We have revised the study design of the manuscript, which we hope will contribute to the understanding of the results. In the first phase (weeks 1-6) participants were randomized into two groups (pre-EOL vs. pre-HS) and in the second phase (weeks 8-13) participants crossed over between the two groups (pre-EOL to post-HS, pre-HS to post-EOL). We also have revised flowchart of the experiment. and re-uploaded it as an attachment.

**33.Response to comment:** *L 263-265:It could be suitable to report the magnitude of the significant changes in the text to allow the reader a better adoption of the findings.*

**Responds:** Thank you for your valuable comments that so enhance the quality of our manuscript, and We have revised the sloppy presentation. We have add the magnitude of the significant changes in the text, and uploaded the statistics in the attachment and hope it will help you to solve the confusion of reading. L 299

**34.Response to comment:** *L 277-278:This third comparison lacks of support here, and should be presented in the statistics section.*

**Responds:** Thank you for your valuable comments that so enhance the quality of our manuscript, and We have made additional revision in the statistics section. In L248

**35.Response to comment:**: *L 282-283:This sentence is misconducting here since the mention of rest interval suggest that post-measurements would be presented, while it seems that comparison is performed on PRE measurements. Please amend and/ or correct.*

**Responds:** We apologize for any confusion caused in the article. Thank you for your valuable comments that so enhance the quality of our manuscript, and We have modified the experimental design. Since the previous presentation of the experimental design caused confusion in your reading of the results. Again, we apologize. In L124-140

**36.Response to comment:** *L 284-285:Please state clearly this difference in the text*

**Responds:** Thank you for your valuable comments that so enhance the quality of our manuscript, and we have completed the changes in the manuscript. We have added relevant information. In L321-323

**37.Response to comment:***L 288-290:Again this sentence is misconducting, since is could be understand that the PRE-HS value is significantly correlated to PPO. Please rephrase.*

**Responds:** We apologize for any confusion caused in the article. Thank you for your valuable comments that so enhance the quality of our manuscript, and We have modified the experimental design. Since the previous presentation of the experimental design caused confusion in your reading of the results. we have re-uploaded the flowchart of the experiment and the statistical results. Again, we apologize. In L124-140

**38.Response to comment:** *L 333-335:How could the authors state that the sprint speed of PRE-EOL could be lower than PRE-HS at 9-min rest interval? Once again this sentence is conflicting here and needs to be rephrase.*

**Responds:** We apologize for any confusion caused in the article. Thank you for your valuable comments that so enhance the quality of our manuscript, and We have modified the experimental design. Since the previous presentation of the experimental design caused confusion in your reading of the results. we have re-uploaded the flowchart of the experiment and the statistical results. Again, we apologize. In L124-140

**39.Response to comment**:*L 342-344: Interactions rather revealed significant differences than* *influenced results. Please rephrase for a better understanding.*

**Responds:**  Thank you for your valuable comments. We have revised the inappropriate statement. Change affect to influence. L380-381

**40.Response to comment:** *L 361-362:It would be suitable to differentiate between stretch shortening cycle occurring during running or CMJ for instance and the succession of eccentric and concentric phase with no (or only a few) storage of passive elastic energy (e.g. squatting).*

**Responds:** We are very grateful for the reviewers' comments. We have added your summary. In L411-413

**41.Response to comment:***L 369-370:This aim differ from the scientific question addressed in the introduction section, and is therefore not supported by the rational. Please ensure consistency about the objectives of this study throughout the manuscript.*

**Responds:** We are very grateful for the reviewers' comments. We have removed the inappropriate expressions. And revised the presentation in the introduction and the discussion section so that the discussion is consistent with the introduction.

**42.Response to comment:***L 398-404:There are contradictory findings mentioned here between improvement in CMJ height and PPO due to EOL training, while non-statistical difference is noted. Please based your statement on the statistical findings and not graphical reading.*

**Responds:** We apologize for any confusion caused in the article. Thank you for your valuable comments that so enhance the quality of our manuscript, and we have revised the presentation in the introduction and the discussion section and modified the experimental design. Since the previous presentation of the experimental design caused confusion in your reading of the results. we have re-uploaded the flowchart of the experiment and the statistical results. Again, we apologize. In L124-140

**43.Response to comment:***L 428-430:This section is quite descriptive and lacks of elements to explain the absence of significant improvement in this study.*

**Responds:** We are very grateful for the reviewers' comments. We have revised the inappropriate statement. In L430-438

**44.Response to comment:***L 438-440:I acknowledge that jumping performance is important for basketball players. However, as it is mentioned previously, the effect of PAPE is only transitory and would not be effective during an entire match.*

**Responds:** We are very grateful for the reviewers' comments. We have revised the description and added a Practical Application section. In L470-477

**45.Response to comment:** *L 447-449:This sentence contradicts the first one of the conclusion section. Please ensure coherence about the presence or absence of PAPE phenomenom in the present study. This question remains unclear while reading this section.*

**Responds:** We are very grateful for the reviewers' comments. We have rewritten the conclusion. In L465-468. We apologize for any confusion caused in the article. Thank you for your valuable comments that so enhance the quality of our manuscript, and We have modified the experimental design. Since the previous presentation of the experimental design caused confusion in your reading of the results. we have re-uploaded the flowchart of the experiment and the statistical results. Again, we apologize. In L124-140

**46.Response to comment:** *L 449-452:Both squatting and flywheel are based on eccentric-concentric contractions here. It appear therefore obvious to precise more specifically the different exercise modalities (e.g.; phase duratin, intensity, ...).*

**Responds:** We are very grateful for the reviewers' comments. We have rewritten the conclusion. In L465-468. We apologize for any confusion caused in the article. Thank you for your valuable comments that so enhance the quality of our manuscript, and We have modified the experimental design. Since the previous presentation of the experimental design caused confusion in your reading of the results. we have re-uploaded the flowchart of the experiment and the statistical results. Again, we apologize. In L124-140

**Response to Reviewer #2:**

**1.Response to comment:**  *Line 46-47:Not clear, rephrase and explain clearly the design.*

**Responds**: We apologize for any confusion caused in the article. Thank you for your valuable comments that so enhance the quality of our manuscript, and We have modified the Methods L 42-51. We have modified the experimental design. Since the previous presentation of the experimental design caused confusion in your reading of the results. we have re-uploaded the flowchart of the experiment and the statistical results. Again, we apologize. In L124-140

**2.Response to comment:** *Lines 49-51: Not useful in an abstract.*

**Responds:** Thank you for your valuable input. We have removed the inappropriate statement.

**3.Response to comment:** *Lines 52-58:There is no consistency between the methods and the results.*

**Responds:**We apologize for any confusion caused in the article. Thank you for your valuable comments that so enhance the quality of our manuscript, and We have modified the result. L52-66 We have modified the experimental design. Since the previous presentation of the experimental design caused confusion in your reading of the results. we have re-uploaded the flowchart of the experiment and the statistical results. Again, we apologize. In L124-140

**4.Response to comment:** *The last sentence makes no sense since every movement is a combination of concentric/eccentric actions*.

**Responds:** Thank you for your valuable input. We have removed the inappropriate statement.

**5.Response to comment:** *Lines 103-106:It is not clear what “long season” means here. Please explain.*

**Responds:** We are very sorry that our presentation has caused you confusion. We intended to say over a season. In L117

**6.Response to comment:***Overall, why half-squat and not other squat variations? Please elaborate.*

**Responds:** We are very sorry that our presentation has caused you confusion. We have modified the expression, the flywheel exercise is a half-squat movement pattern, in order to compare the effect of the two programs, the traditional squat also uses the barbell half-squat pattern.

**7.Response to comment:**: *Line 195:PAP or PAPE?*

**Responds:** We are very grateful for the reviewers' comments. For the reviewers' valuable comments, we add the study by Blazevich and Babault. In this study, the term PAPE was used in relation to the enhancement of muscle contraction after conditioning activity. In L80-81

**8.Response to comment:***The statistical analysis should be rerun: it is not clear to me why two different two-way analysis were performed instead of a three-way.*

**Responds:** Thank you for such valuable comments, and we have asked several statistical experts to conduct a thorough evaluation of the statistical methods in the manuscript, and they believe that we have a cross-sectional design, however a repeated measures design nested within it that measures outcome indicators at different time points, so the two-factor ANOVA and single-cause repeated measures ANOVA used in the current manuscript support our conclusions well.

**9.Response to comment:**: *This is a major point that should be addressed. Therefore, I have stopped my review here, since the results should be rewritten accordingly.*

**Responds:** For this, I am very sorry. We have made many revision changes to address the issues you have raised. Please continue to review our manuscript. Thank you.

**Response to Reviewer #3:**

**1.Response to comment:** *The manuscript refers to post-activation potentiation (PAP) throughout – attributed to phosphorylation of myosin regulatory light chains.*

**Responds:** We are very grateful for the reviewers' comments. After carefully reading the reviewers' comments, We adopted the concept of PAPE, complemented the relevant literature, and described the mechanisms associated with PAP and PAPE. In L83-90.

**2.Response to comment:** *The terms used to describe the exercises are quite vague. For example, an eccentric overload could be achieved in many different ways and a ‘loaded half squat’ could also be achieved in different ways. In fact, it could be argued that both exercises are ‘loaded half squats.*

Responds: We are very grateful for the reviewers' comments. We have carefully reviewed and rewritten the methods. We compare flywheel eccentric overloading and barbell squatting.

**3.Response to comment:**  *The loads used for the barbell squat are individual-specific (a percentage of 1RM) but the flywheel inertias are not. This warrants further consideration or discussion.*

**Responds:** We are very grateful for the reviewers' comments. We acknowledge that comparing the effects of two different movement patterns is a great challenge, and we have discussed it. In L402-410

**4.Response to comment:** *In the statistical analysis section, it is stated that one-way repeated measures ANOVAs were performed. However, a few lines later on talk about interaction effects, which suggest that at least a two-way ANOVA was performed.* **Responds:** Thanks to such valuable comments, we have rephrased the study design and uploaded the study roadmap with the results of data analysis. We have also considered carefully and invited statistical experts for a thorough review of the study methodology in the manuscript. However, they felt that they believed that we were a cross-sectional design and, with a repeated measures design nested within it that measured outcome indicators at different time points, and therefore, suggested that a two-factor ANOVA and a single-cause repeated measures ANOVA would support our conclusions well in the manuscript.

Although we did not make the assumption that the two minimum strengths (flywheel and barbell) are equal, we standardized the movement pattern of the half squat (flywheel and barbell) and We used the lightest weight and inertia torque of the two devices as the low intensity for this test. We also consider this a great challenge.

**5.Response to comment:** *Specific comments:Authors: It is not clear how there can be three co-first authors.*

Responds: We have revised to two co-first authors.

**6.Response to comment:** *Short title: Compared to what?*

**Responds:** We are very grateful for the reviewers' comments. Flywheel half-squat compare to barbell half-squat exercise.

7**.Response to comment:** *Data availability statement: I suggest that you upload the data alongside the manuscript for readers and reviewers in line with journal policy.*

**Responds:** We are very grateful for the reviewers' comments. We have uploaded the statistics rusult in the attachment.

**8.Response to comment:** *Line 52:PPO should be defined. Peak power output? It would also be useful to include some statistical results within the abstract.*

Responds: We greatly appreciate the reviewers' comments. We have changed the definition of PPO to Peak power output (PPO). In L52

**9. Response to comment:** *Lines 74-75:This statement is too dramatic/ambitious and should be reduced in everity.*

**Responds:** We greatly appreciate the reviewers' comments. We have modified the inappropriate statement. In L80-93

**10. Response to comment:***Line 95:Other studies have investigated the relationship between flywheel moment of inertia and velocity or power (concentric and eccentric) during flywheel half squats – see https://doi.org/10.1080/02640414.2020.1860472*

**Responds:** We greatly appreciate the reviewers' comments. We have added and revised the presentation in the preface and discussion sections, and added references.

**11. Response to comment:** *Lines 101-106:Although not on jumping sports, quite a few PAPE studies have focused on vertical or horizontal jumps.*

**Responds:** We are very grateful for the reviewers' comments. As the presentation has been rewritten, we have added relevant references in different places.

**12. Response to comment:** *Lines 118-120:It’s unclear how many sessions this is in total. How many familiarisation sessions were performed if any?*

**Responds:** We are very grateful for the reviewers' comments. 12 sessions in total, an additional 1 familiarization sessions were performed, and we have cited references and modified the presentation accordingly. The corresponding statement was modified in the method section. We have modified the experimental design. Since the previous presentation of the experimental design caused confusion in your reading of the results. we have re-uploaded the flowchart of the experiment and the statistical results. Again, we apologize. In L124-140

**13. Response to comment:**  *Line 148:Why 12 participants? This number should be justified. See https://doi.org/10.1525/collabra.33267*

**Responds:** Thank you for your valuable comments that so enhance the quality of our manuscript, and we have the changes in the manuscript. The entire membership of a basketball team participated in the trial, originally 18, with 6 withdrawing due to injury. In L260-265

**14. Response to comment:***Line 161 and elsewhere:I suggest being consistent with ‘participants’ or ‘subjects’ – the section is called ‘Participants’ so it would be good to stick with this.*

**Responds:** Modified to: participants.

**15. Response to comment:** *Lines 178 and 188:Why were two trials averaged? Why two and why an average?*

**Responds:** We are very grateful for the reviewers' comments. We have revised the presentation. Two tests were conducted to ensure that it was a maximum effort and that the results met the criteria of the movement pattern. The best performance was used for statistical analysis. In L188 and L202

**16. Response to comment:** *Lines 185-186:How were these parameters calculated? There are multiple possible methods for some of them.*

**Responds:** Thank you for your valuable comments. The jump performance parameters were measured by Smart Jump (Fusion Sport; Australia) and standardized to the CMJ movement pattern. In L193-195

**17. Response to comment:** *Line 192:What was the starting position for the sprint? In line with the timing gate or slightly behind?*

**Responds:** We are very grateful for the reviewers' comments. We have revised the presentation. In L207-209

**18. Response to comment:***Line 201:Why 3 sets x 6 reps? The volume PAPE paper mentioned earlier could be used here, but it is important to justify these choices.*

**Responds:** Thank you for your valuable comments that so enhance the quality of our manuscript. The exercise was performed by reference: 10.1519/JSC.0000000000003005. The applicability of the method will be studied in depth subsequently. Maroto-Izquierdo et al. (10.5114/biolsport.2020.96318) found that a single set (i.e., 6 repetitions) of the half-squat flywheel exercise performed with the optimal concentric peak power intensity in physically active young men led to significant acute gains in CMJ jump height.

**19 Response to comment:***Lines 208-209: Were participants instructed to resist throughout the eccentric phase or only in a certain portion of it?*

**Responds:** Thank you for your valuable comments that so enhance the quality of our manuscript, and we have revised in the manuscript. Eccentric process is resisted, knee flexion control at 90 degrees as the limit for centripetal contraction. In L219-224

**20. Response to comment:***Lines 232-233:These should be reworded with signs such as ‘greater than or equal to’ to ensure that there are no gaps. For example, 1.195 and 1.995 currently have no category.*

**Responds:** Thank you for your valuable comments. We have rephrased trivial (<0.200), small (0.200 - 0.599), moderate (0.600 - 1.199) and large (1.200 -1.999), very large (≥2.000). In L254-255

**21. Response to comment:***I suggest adding tables to make the results clearer.*

**Responds:** Thank you for your valuable comments. We have added the statistical result in the attachment.

**22. Response to comment:***Line 368:‘shown’ may be better than ‘proved’ – to show more uncertainty.*

**Responds:** Thank you for your valuable comments. We have revised the inappropriate statement.

**23. Response to comment:** *It would be good to re-summarise the main overall results early in the Discussion.*

**Responds:** Thank you for your valuable comments. We have rewritten the main overall results early in the Discussion. In L397-401

**24. Response to comment:***Lines 369-370: More justification is needed for why basketballers might be different to other populations and why results might not continue over a season.*

**Responds:** Thank you for your valuable comments. We have added Practical Applications. In L 470-477

**25. Response to comment:**  *Line 382:What is meant by ‘ground-lifting’?*

**Responds:** Thank you for your valuable comments. Due to inaccurate translation, we have removed the expression.

**26. Response to comment:** *Lines 384-387:Some of the studies I mentioned above (e.g. the effects of inertia on PAPE) may be useful here. Likewise, for lines 390-391 where the differential effects of peak power / velocity / force are discussed.*

**Responds:** Thank you for your valuable comments. We have revised the presentation and cited the relevant literature. In L 418-423

**27. Response to comment:** *Line 397: Could this be controlled for within the statistical analysis?*

**Responds:** Thank you for your valuable comments. We did make relevant controls, re-upload the study roadmap and data statistics, and rewrote the study design to make it easier to understand.

**28. Response to comment:***Line 411:Was this ‘tendency’ significant? If not then it should not be discussed as an effect. The same applies in line 448 (if not then this should not be part of the conclusion).*

**Responds:** Thank you for your valuable comments. We have revised the word to effect in the relevant position.

**29. Response to comment:** *Lines 418/420/421-consistency needed around minutes / -minutes / min*

**Responds:** Thank you for your valuable input. For consistency of presentation, we have modified the expression to read: min.

We gratefully appreciate for your valuable suggestion

We tried our best to improve the manuscript and made some changes in the manuscript. These changes will not influence the content and framework of the paper. And all the changes are marked in red in revised paper.

We appreciate for Editors/Reviewers’ warm work earnestly, and hope that the correction will meet with approval.

Once again, thank you very much for your comments and suggestions.

Yours sincerely,

Jian Sun, School of Athletic Training, Guangzhou Sport University, Guangzhou, P.R. China, E-mail: [sunjian@gzsport.edu.cn](mailto:sunjian@gzsport.edu.cn) . Tel:+8613728059899
