# Supplementary material for: Flywheel eccentric overload exercises versus barbell half squats for basketball players: Which is better for induction of post-activation performance enhancement?
Source: PLoS One. 2022 Nov 21;17(11):e0277432. doi: 10.1371/journal.pone.0277432 (PMC9678314; doi:10.1371/journal.pone.0277432)
Supplement: Attachment — Submitted filename: Responds to Reviewers.docx [file pone.0277432.s003.docx]

Dear editor and dear reviewers

Regarding the manuscript ID (NO.PONE-D-22-02783) entitled " Eccentric overload exercises versus loaded half squats for basketball players: which is better for induction of postactivation potentiation?". Thank you for your letter and the reviewers' comments on our manuscript entitled " Eccentric overload exercises versus loaded half squats for basketball players: which is better for induction of postactivation potentiation?" (NO.PONE-D-22-02783) were evaluated. These comments were valuable and helpful to us in revising and improving the paper, as well as important guidance for our research. We have carefully studied these comments and made revisions, which we hope will be approved by all of you. The revised parts are marked in red in the thesis. The main corrections in the paper and the responses to the reviewers are listed below.

**Reply to the first reviewer as follows:**

Question#1: Abstract: L 53: I suggest the authors to precise what time points are considered here? Is it only immediately after exercise, or at all time points? The sentence is misconducting.

Responds: Thank you very much for your valuable advice. We have made changes to the relevant content.Please see L53.

Question#2: L60-62: the use of stage I and II is unclear here, and does not refer to one of the objectives stated previously. I suggest to remove this information in the abstract to make it clearer.

Responds: Thank you for your valuable advice. We have removed this information from the Abstract.

Question#3: L 64-65: this sentence is unclear. If no change on sprint speed was observed after the two training protocols, therefore there was no effect. Please rephrase to make this sentence less confuse.

Responds: Thank you for your valuable advice. We have made changes to the relevant content.Please see L65-66.

Question#4: L 70: would you mean FEOL training?

Responds: Thanks for the tip. We have made changes to the relevant content.Please see L73.

Question#5: Introduction: L 79: I suggest to consider rephrasing into “enhancement in explosive sport performance”

Responds: We have made changes to the relevant content.Please see L82.

Question#6: L 93: replace for “contractility” or “contractile function” depending on the meaning of your sentence

Responds: Thank you for your valuable advice. We have made changes to the relevant content. Please see L96.

Question#7: Methods: L 130: “and in stage-II, they crossed over…”

Responds: Thank you for your prompt. We have revised the relevant content. Please see L135-136.

Question#8: L 135: “were set before training, …”

Responds: Thank you for your tip. We have revised the relevant content. Please see L140.

Question#9: L 169: it is unclear whether the 6 players dropped out because of injuries occurring during the experimental protocol, or were not included because of the inclusion criteria (line 158). Please be consistent throughout the paragraph.

Responds: Thank you very much for your valuable advice. We have revised the relevant content. Please see L164.

Question#10: L 179: as mentioned in a previous revision, the InBody 370 is a body composition analyzer, and could therefore not measure participant’s height. Please indicate the scale used to measure this variable.

Responds: Thank you very much for your valuable advice. We have revised the relevant content. Please see L184-185.

Question#11: L 198: words are lacking here, following what?

Responds: Thank you for your suggestion. We have changed the inappropriate expression. Please see L204-206.

Question#12: L 224: “On the GO signal” could be better

Responds: Sorry. We have made changes to the inappropriate statements. Please see L232.

Question#13: Results: The same problem still remains in this third version of the manuscript about interpretation of the statistical analysis. Authors should carefully translate their result, and mentioned difference, increase or decrease when statistics prove it. When no statistical difference is findings, therefore there is no increase or decrease of the variables, even if the numbers are not the same. This misconducting makes the results fallacious and decrease the quality of the manuscript. A real effort should be provided here to describe clearly and decently these findings.

Responds: We have revised it according to your valuable suggestion. Please see L56-60, L324-329, L340-342, L344-349, L359-362, L364-366, L373-375, L390-392, L412-415, L423-425, and L430-431.

Question#14: Table 1: “indicates significant difference” or “differ significantly “. Under the present form this sentence is unclear

Responds: We have revised it according to your valuable suggestion. Please see Table 1.

Question#15: Discussion

L 422-426: the summary of the main findings is unclear. The first sentence states that no performance improvement was noted for CMJ and sprint after the two protocols. The second sentence argues that FEOL training can increase jump height to a greater extent that BHS. These sentences, under the present form, are therefore contradictory. Would the authors say that jump heights at 3, 6 9 and 12 min were greater for FEOL than BHS? If yes, then rephrase to correspond.

Responds: We have revised it according to your valuable suggestion. Please see L444-446.

Question#16: L 434-435: please state clearly how this performance could be impacted? Improvement? Decrease?

Responds: We apologise for our inappropriate statement. This part is speculation on our part and we are not sure whether these factors actually have an impact on sports performance. What we were actually trying to convey was the advantage of the current crossover design. We have amended this. Please see L452-458.

Question#17: L 450: as state previously for results section, in absence of statistical difference, no difference exists. Please correct to correspond.

Responds: Thank you for your valuable advice. We have revised the relevant content. Please see L311, and L470-471.

**Reply to the third reviewer as follows:**

Question#18: Thank you for your response regarding the low/medium/high intensity for each exercise. I understand that they are not intended to be equivalent intensities between exercises. However, your use of an ANOVA may dictate that they are considered to be equal. For example, does this statistical model assume that you have 3 intensities (low/medium/high) at each of 2 exercises, and then compare? The main and especially the interaction effects may depend on this assumption, so it is worth stating/discussing. If this test is not performed then it is less of an issue, but perhaps giving the exact intensity in tables and results would be better than referring to two different ‘low’, two different ‘medium’, etc. is they are not equivalent.

Responds: Thank you for asking the rigorous questions. At the outset of the design, it was noted that the comparison of the two devices for the semi-squat exercise would be the biggest challenge of this thesis. We have categorised the intensity according to the flywheel rotational inertia attached to the equipment as low (0. 015 kg∙m2), medium (0.035 kg∙m2) and high (0.075 kg∙m2), referencing the Petersen J,et al.[1] literature. In addition, the intensity was adjusted to low (40%), medium (60%) and high (80%) according to the number of sets, repetitions and individual 1RM, Gourgoulis V,et al.[2] ,Fukutani A.[3] ,Buscà B,et al.[4] , and other literature. We agree with you and do assume that they are equal, but in reality they are not. We will follow your suggestion to note the corresponding strengths in numerical form for modification.Please see L63-65, L68-69, L125-126, L142-143, L307-309, L315-317, L319-324, L338-339, L364, L386-389, L394-396, L419-421, L489-491, L521.

[1]Petersen J, Thorborg K, Nielsen MB, Budtz-Jørgensen E, Hölmich P. Preventive effect of eccentric training on acute hamstring injuries in men's soccer: a cluster-randomized controlled trial. Am J Sports Med. 2011 Nov;39(11):2296-303. doi: 10.1177/0363546511419277. Epub 2011 Aug 8. PMID: 21825112.

[2] Gourgoulis V , Aggeloussis N, Kasimatis P, et al. Effect of a submaximal half-squats warm-upprogram on vertical jumping ability[J]. J Strength Cond Res, 2003, 17(2):342-344. doi: 10.1519/1533-4287(2003)017<0342:eoashw>2.0.co;2

[3] Fukutani A, Takei S, Hirata K, et al. Influence of the intensity of squat exercises on thesubsequen jump performance[J]. J Strength Cond Res, 2014, 28(8):2236-2243. doi:10.1519/JSC.0000000000000409.

[4] Buscà B, Aguilera-Castells J, Arboix-Alió J, Miró A, Fort-Vanmeerhaeghe A, Peña J. Influence of the Amount of Instability on the Leg Muscle Activity During a Loaded Free Barbell Half-Squat. Int J Environ Res Public Health. 2020 Oct 31;17(21):8046. doi: 10.3390/ijerph17218046. PMID: 33142906; PMCID: PMC7672597.

Question#19: Please check your equations in lines 210-212. I get different answers using these compared to standard equations. It is also not clear to me why flight time is divided by 1000 (instead of 2). If it is recorded in ms instead of s then this should be clear. I suggest indicating that the PPO is an estimate and not a measurement. If possible, I also suggest using g as 9.81 instead of 9.8.

Responds: Thank you for your valuable advice. We have specified the unit ms in flight time and have used the PPO as an estimate rather than a measurement as you suggested, which has been very enlightening. Also changed the gravity (g) to 9.81 m/s. Please see L216-219.

We gratefully appreciate for your valuable suggestion

We tried our best to improve the manuscript and made some changes in the manuscript. These changes will not influence the content and framework of the paper. And all the changes are marked in red in revised paper.

We appreciate for Editors/Reviewers’ warm work earnestly, and hope that the correction will meet with approval.

Once again, thank you very much for your comments and suggestions.

Yours sincerely,

Jian Sun, School of Athletic Training, Guangzhou Sport University, Guangzhou, P.R. China, E-mail: [sunjian@gzsport.edu.cn](mailto:sunjian@gzsport.edu.cn) . Tel:+8613728059899
