# Supplementary material for: Flywheel eccentric overload exercises versus barbell half squats for basketball players: Which is better for induction of post-activation performance enhancement?
Source: PLoS One. 2022 Nov 21;17(11):e0277432. doi: 10.1371/journal.pone.0277432 (PMC9678314; doi:10.1371/journal.pone.0277432)
Supplement: Attachment — Submitted filename: Responds to Reviewers.docx [file pone.0277432.s004.docx]

**Dear editor and dear reviewers**

Regarding the manuscript ID (NO.**PONE-D-22-02783**) entitled " Eccentric overload exercises versus loaded half squats for basketball players: which is better for induction of postactivation potentiation?". Thank you for your letter and the reviewers' comments on our manuscript entitled " Eccentric overload exercises versus loaded half squats for basketball players: which is better for induction of postactivation potentiation?" (NO.PONE-D-22-02783) were evaluated. These comments were valuable and helpful to us in revising and improving the paper, as well as important guidance for our research. We have carefully studied these comments and made revisions, which we hope will be approved by all of you. The revised parts are marked in red in the thesis. The main corrections in the paper and the responses to the reviewers are listed below.

**Comments to the Author**

1. If the authors have adequately addressed your comments raised in a previous round of review and you feel that this manuscript is now acceptable for publication, you may indicate that here to bypass the “Comments to the Author” section, enter your conflict of interest statement in the “Confidential to Editor” section, and submit your "Accept" recommendation.

Reviewer #1: All comments have been addressed

Reviewer #3: (No Response)

2. Is the manuscript technically sound, and do the data support the conclusions?

The manuscript must describe a technically sound piece of scientific research with data that supports the conclusions. Experiments must have been conducted rigorously, with appropriate controls, replication, and sample sizes. The conclusions must be drawn appropriately based on the data presented.

Reviewer #1: (No Response)

Reviewer #3: Yes

3. Has the statistical analysis been performed appropriately and rigorously?

Reviewer #1: (No Response)

Reviewer #3: Yes

4. Have the authors made all data underlying the findings in their manuscript fully available?

The PLOS Data policy requires authors to make all data underlying the findings described in their manuscript fully available without restriction, with rare exception (please refer to the Data Availability Statement in the manuscript PDF file). The data should be provided as part of the manuscript or its supporting information, or deposited to a public repository. For example, in addition to summary statistics, the data points behind means, medians and variance measures should be available. If there are restrictions on publicly sharing data—e.g. participant privacy or use of data from a third party—those must be specified.

Reviewer #1: (No Response)

Reviewer #3: Yes

5. Is the manuscript presented in an intelligible fashion and written in standard English?

PLOS ONE does not copyedit accepted manuscripts, so the language in submitted articles must be clear, correct, and unambiguous. Any typographical or grammatical errors should be corrected at revision, so please note any specific errors here.

Reviewer #1: (No Response)

Reviewer #3: Yes

6. Review Comments to the Author

Please use the space provided to explain your answers to the questions above. You may also include additional comments for the author, including concerns about dual publication, research ethics, or publication ethics. (Please upload your review as an attachment if it exceeds 20,000 characters)

Reviewer #1: (No Response)

Reviewer #3: My previously recommended revisions have again mostly been addressed. I have one comment remaining which I believe represents a major assumption within the manuscript. The authors recognise this but it should be made clear throughout the article.

You have confirmed within your response that the statistical analysis treats both exercises as having equal low, medium, or high resistance. The results are therefore inherently based on the degree to which the intensities are well matched. You recognised this within your reviewer response, but I believe it should be made clear throughout all sections of the manuscript so that (potentially uninformed) readers can make their own interpretation of the results based on this assumption.

**Responds:** We agree with you so much that we have revised the text. Please see L138-140, L486-490.
